# Non-equilibrium crystallization pathways of manganese oxides in aqueous solution

**DOI:** 10.1038/s41467-019-08494-6

**Published:** 2019-02-04

**Authors:** Wenhao Sun, Daniil A. Kitchaev, Denis Kramer, Gerbrand Ceder

**Affiliations:** 1 Materials Sciences Division, Lawrence Berkeley National Laboratory, Berkeley, CA 94720 USA; 2 Department of Materials Science and Engineering, Massachusetts Institute of Technology, Cambridge, MA 02139 USA; 3 Engineering Sciences, University of Southampton, Southampton, SO17 1BJ UK; 4 Department of Materials Science and Engineering, University of California, Berkeley, CA 94720 USA

## Abstract

Aqueous precipitation of transition metal oxides often proceeds through non-equilibrium phases, whose appearance cannot be anticipated from traditional phase diagrams. Without a precise understanding of which metastable phases form, or their lifetimes, targeted synthesis of specific metal oxides can become a trial-and-error process. Here, we construct a theoretical framework to reveal the nanoscale and metastable energy landscapes of Pourbaix (*E-p*H) diagrams, providing quantitative insights into the size–dependent thermodynamics of metastable oxide nucleation and growth in water. By combining this framework with classical nucleation theory, we interrogate how solution conditions influence the multistage oxidation pathways of manganese oxides. We calculate that even within the same stability region of a Pourbaix diagram, subtle variations in pH and redox potential can redirect a non-equilibrium crystallization pathway through different metastable intermediates. Our theoretical framework offers a predictive platform to navigate through the thermodynamic and kinetic energy landscape towards the rational synthesis of target materials.

## Abstract (web summary)

Multistage crystallization of transition metal oxides often proceeds through a number of metastable intermediates. Here, the authors shed light on this phenomenon by extending Pourbaix diagrams to capture the metastable and nanoscale thermodynamics of manganese oxide nucleation.

## Introduction

Transition metal oxides drive the functionality of an enormous range of technological materials; spanning battery cathodes, catalysts, fuel cells, magnetic media, and more. The breadth of transition metal oxide applications largely stems from the diversity of their electronic, optical, and magnetic properties, which can be tuned as a function of the crystal structure and metal oxidation state^1^. Understanding how to rationally synthesize metal oxides in desired phases, with desired oxidation states, is central towards unlocking the full potential of transition metal oxide design. The manganese oxides are a remarkable example of structural and oxidation-state diversity, spanning more than 30 phases over oxidation states from Mn^2+^ to Mn^7+^^2^. This broad structural diversity makes manganese oxides relevant for a variety of applications; for example, the spinel λ-MnO_2_ phase is an important lithium-ion battery cathode^3^; ramsdellite-MnO_2_ is used in alkaline batteries^4^; and Mn^3+^ containing phases, such as Hausmannite Mn_3_O_4_ and bixbyite Mn_2_O_3_, are precursors to water-splitting catalysts^5–8^. The precipitation and dissolution of various manganese (oxy)hydroxides also play important roles in redox-active biogeochemical processes, such as mediating oceanic O_2_/H_2_S cycles^9^, microbial metabolic cycles^10^, and soil chemistry^11^. Unfortunately, the structural diversity of the manganese oxides also results in a myriad of possible crystallization pathways in solution, which often leads to poor phase-control during crystal growth. Although synthesis recipes to specific manganese oxide phases have been identified and cataloged^5,12^, a comprehensive understanding of the thermodynamic and kinetic processes that drive phase-selection during aqueous crystallization remains elusive.

Previously, we found that spectator ions can be important MnO_2_ structure-directing agents, as intercalation of aqueous alkali cations such as Li^+^, Na^+^, K^+^, etc. can stabilize the metastable α-MnO_2_, δ-MnO_2_, and λ-MnO_2_ polymorph frameworks at off-stoichiometric compositions^13^. However, precipitation of manganese oxides often proceeds by Ostwald’s ‘Rule of Stages’^14^, where a variety of metastable manganese oxides and oxyhydroxides nucleate and grow prior to the formation of the equilibrium phase^15,16^. These non-equilibrium crystallization pathways can occur both with^17,18^, and without^19^ impurity ions in solution. Further complicating the matter, small variations in precursor choice and solution redox conditions can change which metastable phases are observed, as well as their lifetimes, even when the final equilibrium product remains unchanged^20^. Understanding how solution chemistry influences structure-selection along a non-equilibrium crystallization pathway would enable the rational design of aqueous synthesis routes; either towards desirable metastable phases, or away from long-lived metastable byproducts and towards the synthesis of a desired equilibrium phase^21,22^. Importantly, a predictive understanding of hydrothermal synthesis developed on the manganese oxides could be broadly generalized and applied to other transition metal oxide systems.

According to classical nucleation theory, a metastable phase can precipitate first from a supersaturated solution if it has a lower nucleation barrier than the stable phase^23–25^, The nucleation barrier takes the form:

1 $$\Delta G_{\mathrm{c}} \propto \frac{{\left( {\eta \gamma } \right)^3}}{{( - RT\ln \sigma )^2}}$$

where *ηγ* is the shape-averaged surface energy, $\Delta G_{{\mathrm{Bulk}}} = - RT\ln \sigma$ is the bulk thermodynamic driving force for crystallization, and σ is the supersaturation. A metastable phase exhibits a smaller Δ*G*_Bulk_ for its formation than the stable phase, but the metastable phase can still dominate the kinetics of nucleation if this smaller driving force is further compensated by a lower surface energy^26–28^. Calorimetry experiments have shown that many bulk metastable polymorphs have lower surface energies than their corresponding stable phases^29^, and that surface energy differences between divalent (MO), trivalent (M_2_O_3_), and spinel (M_3_O_4_) metal oxides can shift redox equilibria at the nanoscale by orders of magnitude in oxygen fugacity^30^. Incorporating the influence of surface energies on solid-aqueous equilibria at the nanoscale is therefore central towards rationalizing the non-equilibrium crystallization pathways of transition metal oxides in water.

In this work, we extend *E-*pH diagrams, also known as Pourbaix diagrams, to capture the size-dependent thermodynamics of metastable oxide nucleation and growth. First, we construct a thermodynamic grand potential for a metal oxide being acted upon by an external water reservoir with given pH and redox potential, which provides a free-energy axis to Pourbaix diagrams. This free-energy axis is then generalized to incorporate surface energies, enabling the construction of size-dependent Pourbaix diagrams, which capture how particle size influences solid-aqueous equilibria at the nanoscale—where nucleation initiates. The Pourbaix free-energy axis also visualizes how changes in *E* and pH shift the metastable energy landscape, altering the bulk thermodynamic driving forces between reactant and product phases. By combining the Pourbaix potential with classical nucleation theory, we show that even when crystallization starts from the same precursor and ends with the same equilibrium phase, minor variations in *E* and pH can qualitatively change which metastable phases form on the crystallization pathway. Our theoretical framework offers a predictive platform to map how experimental parameters influence non-equilibrium crystallization pathways in redox-active systems, and represents an important step towards a predictive theory of materials synthesis.

## Results

### A thermodynamic grand potential for Pourbaix diagrams

Traditionally, Pourbaix diagrams are constructed by using the Nernst equation to calculate *E*-pH boundaries between aqueous phase stability regions^31^. However, Pourbaix diagrams constructed by this approach do not have a free-energy axis, which makes it challenging to incorporate surface energies and other forms of thermodynamic work into solid-aqueous stability analyses. Adding a free-energy axis for Pourbaix diagrams can also facilitate the evaluation of thermodynamic driving forces between precursors and crystallization products^32^, for example, when a Mn^2+^(aq) precursor is under *E-*pH conditions where it is metastable with respect to the nucleation of solid MnO_2_. To add a free-energy axis to Pourbaix diagrams, here we use a thermodynamic grand potential^33,34^, that corresponds to an aqueous ion precursor or metal oxide precipitate in open exchange with a water reservoir at a given pH, redox potential, and dissolved metal ion concentration. Because these are the natural variables of the Pourbaix diagram, we refer to this thermodynamic grand potential as the Pourbaix potential.

We construct the Pourbaix potential, Ψ, by a Legendre transformation of the Gibbs free energy with respect to the oxygen chemical potential, *µ*_O_; the hydrogen chemical potential, *μ*_H_; and redox potential, *E*, under a constraint of water-oxygen equilibrium. Details of this Legendre transformation can be found in the Methods section, and the final expression for the Pourbaix free energy, normalized by number of metal atoms, is expressed as:

2 $$\begin{array}{ccccc}\\ \overline \Psi = \frac{1}{{N_{Mn}}}\left( {\left( {G - N_O\mu _{H_2O}} \right) - {\mathrm{RT}} \cdot \ln (10) \cdot \left( {2N_O - N_H} \right)p{\mathrm{H}}} \right.\\ \left. { - \left( {2N_O - N_H + Q} \right)E} \right)\\ \end{array}$$

Where *G* is the molar Gibbs formation free energy; *N*_M_, *N*_O_, and *N*_H_ is the composition of the metal oxide/ion; and if the phase is an aqueous ion, the charge, *Q*, normalized by e^−^ per formula unit. Using the Pourbaix potential, the relative free-energies between metal-containing phases of different compositions can be compared directly, without needing to explicitly evaluate redox reactions.

To apply this grand potential to the Mn–H_2_O system, we use the thermochemical dataset shown in Table 1. Although it is possible to compute Gibbs formation energies for both aqueous ions and solid-state phases using ab initio methods^35,36,37^, bulk formation free energies for most of the relevant aqueous and solid-state Mn–O–H phases are known experimentally (Supplementary Table 1), which we use in this work. Missing formation energies for the bulk metastable phases *β*-MnOOH, R-MnO_2_, and γ-MnO_2_ are supplemented using DFT-SCAN calculations^38^, which we have previously shown to give an accurate description of the energetic ordering and enthalpy differences between polymorphs for the manganese oxides^39^. Formation energies for these three metastable compounds are obtained by referencing the free-energy difference of the metastable polymorph against the ground-state phase of the same composition. For Feitknechtite *β*-MnOOH, whose structure is not known, we first performed an ab-initio structure prediction by hydrogenating various layered MnO_2_ phases, as shown in Supplementary Figs. 1 and 2, resulting in a structure with good agreement with experimental XRD patterns (Supplementary Fig. 3), and similar to the recently resolved *β*-NiOOH phase (Supplementary Fig. 4)^40^. Further discussion of the *β*-MnOOH structure prediction process is detailed in Supplementary Note 1.

**Table 1:** Thermochemical data for Mn–H_2_O solids

Phase	Δ*G*^*o*^_f_ at 25 °C (eV/formula)	Source	Surface energy (J/m^2^)	Source
Mn_3_O_4_	−13.300	Hem (1983)^20^	0.96	Birkner (2012)^45^
α-Mn_2_O_3_	−9.132	Hem (1978)^60^	1.36	This work
Mn(OH)_2_	−6.381	Hem (1983)^20^	0.47	This work
*β*-MnOOH	−5.670	This work	0.53	This work
α-MnOOH	−5.763	Fritsch (1997)^47^	0.65	This work
γ-MnOOH	−5.780	Hem (1983)^20^	0.84	This work
R-MnO_2_	−4.767	Kitchaev (2015)^39^	1.33	This work
γ-MnO_2_	−4.787	Kitchaev (2015)^39^	1.44	This work
*β*-MnO_2_	−4.821	Hem (1983)^20^	1.55	This work

**Fig. 1:**
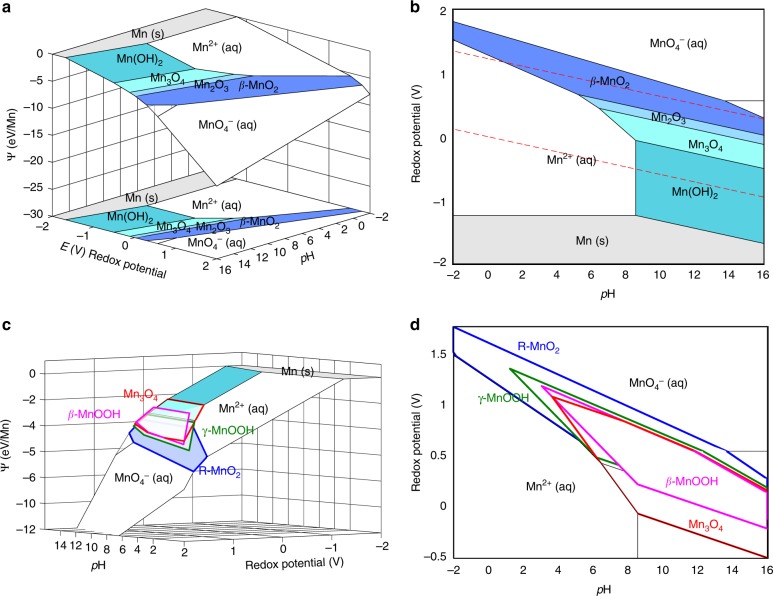
Construction of Mn–H_2_O Pourbaix diagrams using the Pourbaix thermodynamic potential. **a** Concave lowest-energy envelope of Pourbaix free-enesrgy planes in system at [Mn] = 10^−2^ M and 25 °C. **b** Stability regions of equilibrium Mn–O–H phases as projected onto *E-*pH axis. Red dashed lines correspond to redox stability window of water. **c** Pourbaix free-energy planes of metastable β-MnOOH, γ-MnOOH, R-MnO_2_, and full aqueous stability region of Mn_3_O_4_. **d** Projection of aqueous metastability regions onto *E*-pH axis

To construct a Pourbaix diagram, each phase is represented by a Pourbaix free-energy surface, Ψ(*E*,pH); as defined in Eq. (2). Example Pourbaix potentials for the manganese oxide system can be found in Supplementary Note 2. The lowest-energy concave envelope formed by the intersection of all competing free-energy surfaces defines the stable phases and their phase boundaries, as shown in Fig. 1a. By projecting these stability regions onto the *E*-pH plane, the conventional Pourbaix diagram is retrieved, as shown in Fig. 1b. Metastable phases, which do not typically appear on Pourbaix diagrams^31^, can be visualized in Ψ-*E*-pH space, as highlighted in Fig. 1c. By computing the intersection of metastable Pourbaix free-energy planes with the planes of the aqueous ions, one can visualize the full aqueous region where a stable or metastable compound is electrochemically supersaturated. In Fig. 1d, we outline phase boundaries for metastable *β*-MnOOH, *γ*-MnOOH, *R*-MnO_2_, and the full supersaturation region for Mn_3_O_4_. Figure 1d shows numerous regions with overlapping metastable phase boundaries, for example, at conditions corresponding to neutral aerated water (*E* ~ 0.5 V, *p*H ~ 7)^41^. Precipitation of manganese oxides under these conditions would tread the bulk stability regions of numerous thermodynamically-competitive phases.

### Nanoscale Pourbaix diagrams

From bulk energies alone, one cannot distinguish which of the multiple competing metastable phases in Fig. 1d actually precipitates first during crystallization. Calorimetry experiments have shown that metastable oxides can be stabilized at the nanoscale if they have lower surface energy than the equilibrium phases^29^. Because all materials nucleate and grow through the nanoscale, this nanoscale stabilization of metastable oxides should be intimately related to structure selection during materials formation. The Pourbaix potential is a free-energy expression, meaning it can be easily generalized to incorporate surface energies by adding the conjugate variables *γ*A as:

3 $$\overline \Psi (R) = \overline \Psi _{Bulk} + \left( {\frac{1}{R}} \right)\eta \rho \gamma$$

Where *γ* is the surface energy, *R* is an ‘effective’ particle radius—representing the specific surface area in units of Area/Volume, *η* is the unitless shape factor (Area/Volume^2/3^) of the equilibrium particle morphology, and *ρ* is the volume normalized per mole of metal.

**Fig. 2:**
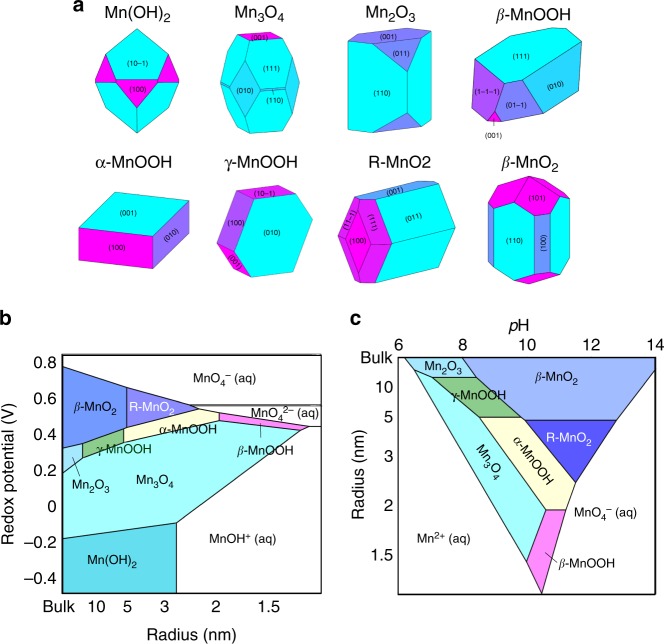
Surface energy contributions to Mn–H_2_O phase equilibria at the nanoscale. **a** DFT-computed Wulff constructions of the equilibrium particle morphologies of size-stabilizable manganese oxide phases. Detailed surface energy data can be found in Supplementary Note 3, and Supplementary Tables 2-11, and Supplementary Figs. 5-14. Wulff constructions are color-coded by the relative surface energies of each facet; blue indicates lower relative surface energy, magenta indicates higher. **b** Size-dependent Pourbaix diagrams at [Mn] = 10^−2^ M and 25 °C, with varying redox potential at fixed pH = 11, and with **c** varying pH, at fixed redox potential *E* = 0.5 V

We compute surface energies for all solid manganese oxide and oxyhydroxides using DFT slab calculations, prepared using the efficient creation and convergence scheme we developed in refs. ^42,^^43^, and computed using the SCAN metaGGA functional^44^. For each phase, we enumerate the low-index surfaces and their unique terminations, which are used in the Wulff construction to determine their equilibrium particle morphologies, as shown in Fig. 2a. The morphology-averaged surface energies for the MnO_x_H_y_ phases are shown in Table 1. Our DFT-computed surface energies of bixbyite Mn_2_O_3_ and pyrolusite β-MnO_2_ are found to be within the error bars of the hydrated surface energies as experimentally measured by Birkner and Navrotsky^45^, providing confidence that DFT can calculate accurate surface energies in the manganese oxide system. Further details on surface calculations and surface energy data can be found in Supplementary Note 3.

The size-dependent Pourbaix potential, Ψ(*E,* pH, 1/R), exists in a four-dimensional thermodynamic space, and can be projected onto the pH–1/R axes at fixed *E*, or the *E–*1/R axes at fixed pH, to construct size-dependent Pourbaix diagrams. To highlight the energetic competition between manganese oxide phases at the nanoscale, Fig. 2 shows two example Mn–H_2_O nanoscale Pourbaix diagrams; one varying in redox potential at a fixed pH = 11, and one varying in pH at a fixed redox potential of *E* = +0.5 V.

Surface energy contributions drive three major effects at the nanoscale. First, because surface energy is always positive, the stability regions of all solid phases shrink relative to the aqueous ions as particle size is decreased. Phases that can be stabilized at small particle sizes will require smaller fluctuations during nucleation to grow beyond the critical nucleation radius. These nanoscale Pourbaix diagrams can therefore be used as proxies to estimate *E-*pH conditions where nucleation and crystal growth initiates most readily. Second, as measured by Birkner and Navrotsky, the surface energies of the stable manganese oxide solids are ordered $\gamma _{{\mathrm{Mn}}_{\mathrm{3}}{\mathrm{O}}_{\mathrm{4}}} < \gamma _{{\mathrm{Mn}}_{\mathrm{2}}{\mathrm{O}}_{\mathrm{3}}} < \gamma _{\beta - {\mathrm{MnO}}_{\mathrm{2}}}$^45^ As particle size radius is reduced, our nanoscale Pourbaix diagrams show an enlargement of the Mn_3_O_4_ stability field and reduction of the *β*-MnO_2_ stability field, effectively corresponding to shifts in oxidation-reduction equilibria at the nanoscale^30^. Previously, the precipitation of non-equilibrium oxides have been attributed to kinetic limitations in the transport of oxygen or electron reactants^16,20,46^. Our size-dependent Pourbaix diagrams show that formation of non-equilibrium phases can also have thermodynamic origins, whereby nanoscale shifts in redox equilibria influence the surface work involved in forming nuclei, in turn modifying the kinetics of nucleation.

Third, as shown in Table 1, we calculate the surface energies of MnOOH polymorphs to be ordered $\gamma _{{\mathrm{\beta - MnOOH}}}{\mathrm{ < }}\gamma _{{\mathrm{\alpha - MnOOH}}}{\mathrm{ < }}\gamma _{{\mathrm{\gamma - MnOOH}}}$, and for the MnO_2_ polymorphs $\gamma _{{\mathrm{R - MnO}}_{\mathrm{2}}} < \gamma _{\gamma {\mathrm{ - MnO2}}} < \gamma _{{\mathrm{\beta - MnO2}}}$. The bulk energies of these phases are ordered in the opposite direction, which lead to the aforementioned polymorph stability crossovers at the nanoscale. These inverse relationships between bulk stability and surface energy within each composition may originate from the fact that a metastable structure has less cohesive energy than a stable phase, which implies a lower energy of cleavage—e.g., a lower surface energy. Interestingly, the MnOOH phases are all calculated to have significantly lower surface energies than the MnO_2_ phases, even in isostructural manganese oxide frameworks, such as between γ-MnOOH/β-MnO_2_, and α-MnOOH/R-MnO_2_^47^. This can be rationalized by the H atoms on the cleaved MnOOH surfaces passivating what would otherwise be broken bonds on the bare isostructural MnO_2_ surfaces. The low surface energies of these MnOOH phases rationalize why MnOOH compounds readily precipitate in solution, even though they are measured by calorimetry to be thermodynamically unstable on the bulk Pourbaix diagrams, as shown in Fig. 1b.

### Crystallization pathways in redox-active systems

Multistage crystallization initiates from a metastable precursor, and cascades down in free-energy to the equilibrium phase by a series of phase transformations. To compute a crystallization pathway using the Gibbs free energy, one would evaluate the most favorable series of downhill reactions through a complicated redox reaction network involving the exchange of hydrogen, oxygen, and electrons^48^. However, under the Pourbaix grand potential, all redox reactions at a given aqueous *E*, pH and ion concentration are evaluated implicitly, meaning one can directly construct a one-dimensional free energy ordering between phases of varying composition. By using the Pourbaix free energy as the electrochemical supersaturation in classical nucleation theory, we arrive at a preliminary theoretical framework to evaluate transition metal oxide crystallization pathways in aqueous solution. Using this framework, we demonstrate that subtle variations in *E* and pH can modify which metastable phases occur on the multistage oxidation pathway of Mn^2+^(aq), even when these reactions occur within the same *β*-MnO_2_ stability region of the Pourbaix diagram.

**Fig. 3:**
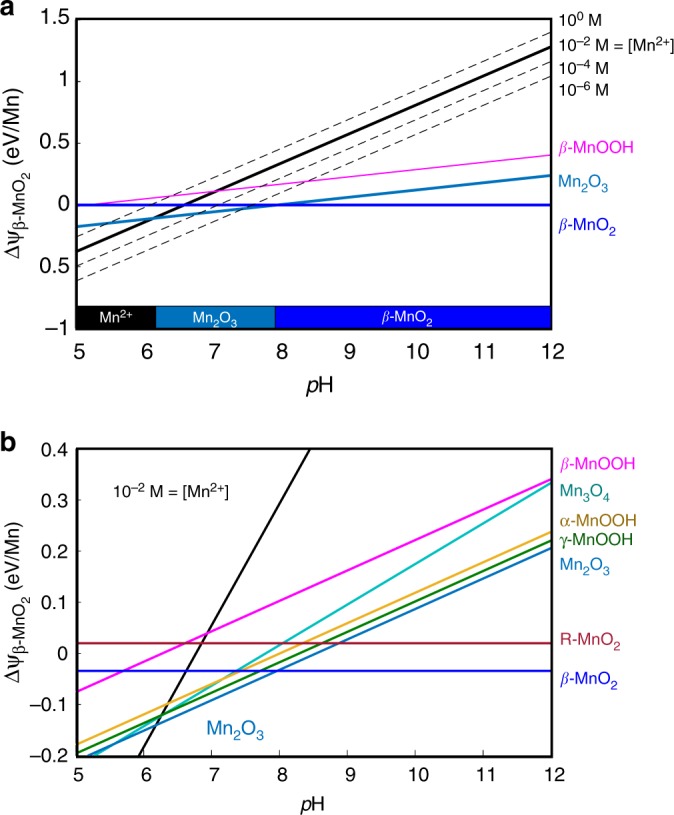
Supersaturation of manganese oxides in solution. Shown for various pH at *E* = 0.5 V. **a** Pourbaix free-energy differences between the Mn^2+^(aq) ion and the *β*-MnO_2_ phase, at varying [Mn^2+^] concentrations (dashed lines). The projection of the lowest free-energy phases onto the pH axis marks the phase stability regions on the Pourbaix diagram. **b** Pourbaix free-energy differences between various solid phases and the *β*-MnO_2_ phase, magnified in the energy axis

Varying *E* and pH within a phase stability region on the Pourbaix diagram does not change the equilibrium phase, but it can shift the metastable energy landscape, altering the thermodynamic driving forces between precursors, intermediates and products. Figure 3 shows a ΔΨ_MnO2_–pH slice of the free energy planes from Fig. 1a, at a fixed *E* = +0.5 V, which is representative of the redox potential in aerated water^41^. The dashed lines in Fig. 3 show that Ψ_Mn2+_ increases linearly with ln[Mn^2+^] concentration, consistent with our traditional intuition regarding supersaturation. At the redox potential shown in Fig. 3, higher [Mn^2+^] activity enlarges the stability region for Mn_2_O_3_, and also increases the supersaturation to *β*-MnO_2_.

However, Fig. 3 also shows that the Mn^2+^(aq) supersaturation is strongly dependent on pH, which is not obvious from a Pourbaix diagram or from ideal solution models. Chemically, a high pH signifies a high concentration of OH^–^ ions, which provides a thermodynamic driving force for the oxidation of Mn^2+^(aq). This oxidation strength can be directly read off of Fig. 3, by the ΔΨ between for example, a metastable Mn^2+^(aq) ion and a metastable MnOOH phase, which increases with increasing pH. Although not shown in Fig. 3, a similar oxidation driving force is achievable through positive redox potentials. Not only do *E* and pH affect the Pourbaix free-energies of aqueous ions, Equation 2 shows that they also affect the relative ΔΨ between solids of different *N*_*O*_ and *N*_*H*_*–*in other words, manganese oxides of different composition. In Fig. 3b, the influence of pH on the free energy differences between the solids Mn_3_O_4_, Mn_2_O_3_, and the polymorphs of MnOOH and MnO_2_ is visualized. Altogether, Fig. 3 reveals a dynamic metastable free-energy landscape over varying electrochemical conditions; a fact that is not readily apparent in traditional Pourbaix diagrams.

**Fig. 4:**
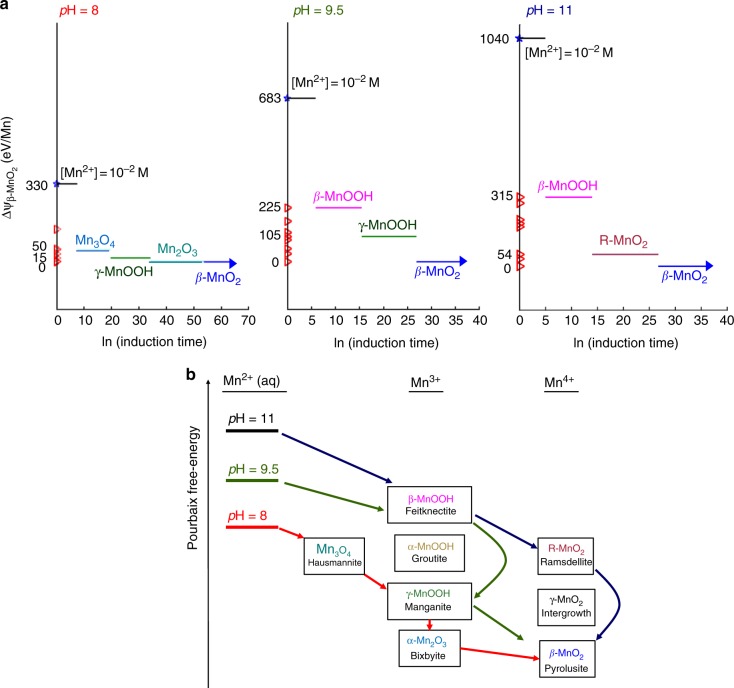
Oxidation pathways of Mn^2+^_(aq)_ at three pH conditions in the *β*-MnO2 stability region. **a** Induction time diagrams, with phases ordered vertically by the ΔΨ of a phase above β-MnO_2_. From the Mn^2+^ precursor, the lowest-barrier phase nucleates and grows, persisting until the induction of the next more-stable phase. The process then repeats. **b** Schematic diagram of the three crystallization pathways as a cascade through the metastable energy landscape; energy axis not drawn to scale

This shifting free-energy landscape can lead to variations in multistage crystallization pathways, even when starting with the same precursor and ending with the same equilibrium phase. In Fig. 4, we construct Pourbaix free-energy orderings of manganese (oxyhydr)oxide phases at three conditions within the equilibrium *β*-MnO_2_ stability window; at pH = 8, 9.5, and 11, with *E* = +0.5 V and [Mn^2+^] = 10^−2^ M. We compute the oxidation pathway of Mn^2+^(aq) under these conditions, using the procedure we derived in ref. ^25^, described here briefly:

Starting from the metastable [Mn^2+^] precursor, we compute the steady-state nucleation rate, *J*, to all lower free-energy phases, by

7 $$J \propto \exp \left( { - \frac{{\left( {\eta \gamma } \right)^3}}{{(\Delta \Psi )^2}}} \right)$$

where the traditional term for supersaturation, $\Delta G = - RT\ln \sigma$, is replaced by the Pourbaix potential, ΔΨ. As shown in the nanoscale Pourbaix diagrams, many of the metastable manganese oxides and oxyhydroxides have lower surface energies than the equilibrium phases. Because the nucleation barrier scales with γ^3^, a low surface energy can compensate for a smaller thermodynamic driving force, ΔΨ, resulting in faster nucleation rates. The induction time of a nucleation event is proportional to the inverse of the nucleation rate, τ ~ 1/*J*, meaning a fast-nucleating metastable phase can grow and consume Mn^2+^ ions prior to the induction of more-stable phases. Even if the metastable phase completes crystal growth by consuming Mn^2+^ to equilibrium, Ψ_Mn2+_ is still supersaturated with respect to the more stable phases. The next lowest-barrier phase nucleates, and this process repeats by dissolution-reprecipitation in a recursive, energetically-cascading series of metastable stages down to the equilibrium phase, β-MnO_2_^25^.

Figure 4a shows the computed Mn^2+^ oxidation pathways under the three considered pH conditions. Near the *β*-MnO_2_ phase boundary at pH = 8, thermodynamic driving forces are small; meaning nucleation barriers are high, induction times are long, and metastable intermediates will be long-lived. From the balance of surface energies and bulk driving forces, we compute a crystallization pathway of Mn^2+^(aq) → Hausmannite Mn_3_O_4_ → Groutite γ-MnOOH → Bixbyite Mn_2_O_3_ → Pyrolusite β-MnO_2_. This progression is qualitatively comparable to the observed crystallization pathways in refs. ^16,^^20^, which proceeded in freshwater at 25 °C over the course of eight months. At higher pH, the increasingly stratified ΔΨ between metastable phases decreases the induction lifetimes of the transient metastable phases, and qualitatively changes the crystallization pathways; forming *β*-MnOOH and bypassing Mn_2_O_3_ at pH = 9.5; and at pH *=* 11, forming *β*-MnOOH and Ramsdellite MnO_2_.

Altogether, these results indicate that hidden above each equilibrium Pourbaix stability region is a complex metastable energy landscape, where free-energy differences between competing phases vary continuously with *E* and pH. As summarized in Fig. 4b, these variations can redirect a crystallization pathway down through different metastable phases, even when crystallization initiates from the same precursor and ends within the same stability region of the Pourbaix diagram. These findings rationalize why non-equilibrium crystallization pathways are so sensitive to solution conditions, and highlight the limitations of using an equilibrium phase diagram to guide materials synthesis^49^.

## Discussion

In this work, we constructed a unified theoretical framework that bridges aqueous electrochemical Pourbaix diagrams, nanoscale crossovers in phase stability, and classical nucleation theory. This combined analysis distinguishes the subtle roles of thermodynamics and nucleation kinetics during the multistage crystallization of redox-active transition metal oxides. In the manganese oxides, we find that surface energy, redox potential and pH all operate on a similar energy scale of manganese oxide metastability^50^, and that all three are important thermodynamic drivers of structure-selection during manganese oxide precipitation. However, the initial precipitation of a non-equilibrium phase can consume much of the reaction driving force, leading to long-lived metastable intermediates and slow nucleation kinetics to the ensuing lower free-energy phases. This delicate balance between thermodynamics and kinetics underlies the complicated dynamics of multistage crystallization, where even in the same phase stability region of a Pourbaix diagram, subtle variations in solution parameters can change which metastable phases a non-equilibrium crystallization pathway passes through. Mapping this metastable energy landscape provides a combined thermodynamic and kinetic foundation for guiding the targeted synthesis of functional metal oxides.

To conclude, we discuss opportunities to achieve more quantitative predictions from this theoretical framework. Is it known that applied pH and redox potential can influence the interfacial structure and adsorption dynamics of the electrochemical double layer^27,51,52^. A more quantitative understanding of the thermochemistry of the electrochemical double layer, especially as a function of surface chemistry and electronic structure, will provide an additional handle to engineer crystallization pathways in solution. Additionally, solid-solid transformations, for example, by H-ion or Mn-ion migration within an oxygen sublattice^53,54^, could be a competing structure transformation mechanism to dissolution-reprecipitation^55^. The energy barriers of ion-diffusion (units of meV/atomic hop) and crystal nucleation (units of meV/nucleus) have different units, and therefore the relative kinetics between these two competing mechanisms cannot be directly compared. A theoretical framework that can treat dissolution-reprecipitation and solid-state transformations on equal footing would enable a more quantitative comparison of the kinetics between diffusive and displacive phase transformations. Finally, we emphasize the need for more deliberate measurements of the redox potential during hydrothermal synthesis, which is an important measure of the oxygen fugacity in water. The redox potential can vary with the concentration of dissolved O_2_ gas, the presence of oxidizing or reducing counterions, and even the relative humidity in air during sample preparation. Careful potentiometric measurements prior to hydrothermal synthesis is necessary to calibrate the thermodynamic redox conditions under which a precipitation reaction is initiated.

## Methods

### A Legendre transform approach to the Pourbaix potential

The Pourbaix potential, Ψ, is a thermodynamic grand potential, and is constructed by a Legendre transformation of the Gibbs free energy with respect to the oxygen chemical potential, *µ*_O_; the hydrogen chemical potential, *μ*_H_; and redox potential, *E*, under a constraint of water-oxygen equilibrium:

8 $$\Psi = G - \frac{{\partial U}}{{\partial N_{\mathrm{H}}}} \cdot N_{\mathrm{H}} - \frac{{\partial U}}{{\partial Q}} \cdot Q - \frac{{\partial U}}{{\partial N_{\mathrm{O}}}} \cdot N_O$$

The partial derivative with respect to charge, *Q*, is the electrical potential, *E*; and the partial derivative with respect to the number of oxygen atoms is the chemical potential of oxygen, *µ*_O_. In solution, the derivative with respect to the number of hydrogen atoms is the chemical potential of a proton *µ*_H_+ at the reference potential (*E* *=* 0 V vs. SHE) minus the electric work *E* required to bring the hydrogen atom into the phase at *E*. The thermodynamic potential can thus be expressed as

9 $$\Psi = G - (\mu _{H^ + } - E)\cdot N_H - E\cdot Q - \mu _O\cdot N_O$$

In an aqueous system, *µ*_O_ and *µ*_H_ + are constrained by the water-oxygen equilibrium

10 $${\mathrm{H}}_{\mathrm{2}}{\mathrm{O}} \leftrightarrow 2 \cdot {\mathrm{H}}^ + + 1{\mathrm{/2}} \cdot \mathrm{O}_2 + 2e^ -$$

which yields

11 $$\mu _{\mathrm{O}} = \mu _{{\mathrm{H}}_{\mathrm{2}}{\mathrm{O}}} - 2 \cdot \mu _{{\mathrm{H}}^{\mathrm{ + }}} + 2E$$

The number of metal atoms are conserved in the phase transformations between metal oxides with different compositions. Thus, Ψ should be normalized by the number of metal atoms. By substituting Eq. M.4 into Eq. M.2 and normalizing by number of metal atoms, we obtain the Pourbaix potential:

12 $$\overline \Psi = \frac{1}{{N_M}}\left( {(G - N_O\mu _{H_2O}) + (2N_O - N_H)\mu _{H^ + } - (2N_O - N_H + Q)E} \right)$$

The molar Gibbs free energy of a phase, *G*, is its chemical potential, *μ*_*i*_ *=* *μ*_*i*_^*o*^ *+* *RT*ln[*a*_*i*_], where *μ*_*i*_^*o*^ is given by the standard-state Gibbs formation free-energy, Δ*G*^*o*^_f_. An ideal solid with no defects has an activity of one, and so the *RT*ln[*a*_*i*_] term is zero, but the chemical potential of metal ions ideally scales with the natural log of the metal ion concentration in solution. The chemical potential of protons can be transformed to pH by the relationship $\mu _{{\mathrm{H}}^{\mathrm{ + }}} = - RT \cdot \ln (10) \cdot {\mathrm{pH}}$.

The Pourbaix potential can be further extended to include the thermodynamic effect of intercalating aqueous impurity ions, as shown in ref. ^13,18^ for intercalating Li^+^, K^+^, Na^+^, Mg^2+^, and Ca^2+^ into various MnO_2_ polymorphic frameworks, by performing another Legendre Transformation on the bulk Gibbs free energy, replacing *G* in Eq. (12) by Φ = *G* – *μ*_M_*N*_M_, where *M* is the relevant aqueous metal ion specie.

### Bulk formation free energies

As shown in Table 1, bulk formation energies are obtained from experimental sources for all phases except *β*-MnOOH, γ-MnO_2_, and R-MnO_2_. For these three metastable compounds, experimental bulk formation free energies do not exist or are not reliable. Formation free energies of these three compounds are obtained from DFT, referenced against their equilibrium polymorphs γ-MnOOH and *β*-MnO_2_. For example,

13 $$\Delta G_{f,\beta {\mathrm{ - MnOOH}}}^ \circ = \Delta G_{f,{\mathrm{\gamma - MnOOH}}}^ \circ + \left( {E_{\beta {\mathrm{ - MnOOH}}}^{DFT} - E_{\gamma {\mathrm{ - MnOOH}}}^{DFT}} \right)$$

Here we assume that the Gibbs free-energy differences between the stable and metastable polymorphs at 298 K are dominated by enthalpy differences. This is a reasonable assumption at room temperatures, where the TΔS term between polymorphic oxides is generally small. The structure of *β*-MnOOH has not been previously reported, so we perform an ab initio structure prediction to resolve this crystal structure, as discussed in Supplementary Note 1.

### DFT calculations

DFT calculations were performed using the Vienna Ab-Initio Software Package (VASP)^56^. We used the projector augmented wave (PAW)^57^ method with the strongly-constrained and appropriately-normed (SCAN)^58^ metaGGA (generalized-gradient approximation) functional. Plane-wave basis cutoff energies were set at 520 eV for all calculations. Brillouin Zones were sampled using Gaussian smearing, with at least 1000 *k-*points per reciprocal atom for bulk unit cells, and at least 700 *k*-points per reciprocal atom for surface slabs. Atoms were initially relaxed until forces were 1E-6 eV/Å. All structure preparations were performed using the Python Materials Genomic (Pymatgen) package^59^.

### DFT surface calculations

Surface energies of MnO_x_H_y_ phases are calculated in density functional theory, using surface slabs generated using the efficient creation and convergence scheme developed by Sun and Ceder^42^. For each conventional bulk unit cell, low-index surfaces are enumerated^43^, and surface energies are calculated within the SCAN metaGGA functional. Surface energy calculations were performed on surface slabs at least 15 Å thick and with 16 Å vacuum. Further details about surface calculations and surface energy data can be found in Supplementary Note 3.

## Supplementary information

Supplementary Information

Peer Review File

## Data Availability

All data necessary to support the findings of this study is available in the manuscript or in the Supplementary Information. Further data and methods can be made available from the authors upon request.

## References

[1] Cox, P. A. *Transition Metal Oxides: An Introduction to Their Electronic Structure and Properties *Clarendon Press (1992).

[2] Post JE (1999). Manganese oxide minerals: crystal structures and economic and environmental significance. Proc. Natl Acad. Sci..

[3] Thackeray MM, Johnson PJ, De Picciotto LA, Bruce PG, Goodenough JB (1984). Electrochemical extraction of lithium from LiMn2O4. Mater. Res. Bull..

[4] Chabre Y, Pannetier J (1995). Structural and electrochemical properties of the proton/γ-MnO2 system. Progress. Solid State Chem..

[5] Robinson DM (2013). Photochemical water oxidation by crystalline polymorphs of manganese oxides: structural requirements for catalysis. J. Am. Chem. Soc..

[6] Stoerzinger KA, Risch M, Han B, Shao-Horn Y (2015). Recent insights into manganese oxides in catalyzing oxygen reduction kinetics. ACS Catal..

[7] Peng H (2017). Redox properties of birnessite from a defect perspective. Proc. Natl Acad. Sci..

[8] Chan, Z. M., et al. Electrochemical trapping of metastable Mn3+ ions for activation of MnO2 oxygen evolution catalysts. *Proc. Natl Acad. Sci*. **115**, E5261-E5268 (2018). 10.1073/pnas.1722235115 PMC6003334 29784802

[9] Tebo BM, Nealson KH, Emerson S, Jacobs L (1984). Microbial mediation of Mn (II) and Co (II) precipitation at the O2/H2S interfaces in two anoxic fjords 1. Limnol. Oceanogr..

[10] Tebo BM (2004). Biogenic manganese oxides: properties and mechanisms of formation. Annu. Rev. Earth Planet. Sci..

[11] Young LB, Harvey HH (1992). The relative importance of manganese and iron oxides and organic matter in the sorption of trace metals by surficial lake sediments. Geochim. Cosmochim. Acta.

[12] McKenzie RM (1971). The synthesis of birnessite, cryptomelane, and some other oxides and hydroxides of manganese. Mineral. Mag..

[13] Kitchaev DA, Dacek ST, Sun W, Ceder G (2017). Thermodynamics of phase selection in MnO2 framework structures through alkali intercalation and hydration. J. Am. Chem. Soc..

[14] Ostwald W (1897). Studien über die Bildung und Umwandlung fester Körper. Z. für Phys. Chem..

[15] Martin, S. T. Precipitation and dissolution of iron and manganese oxides. *Environ. Catal.* **4**, 61–81 (2005).

[16] Murray JW, Dillard JG, Giovanoli R, Moers H, Stumm W (1985). Oxidation of Mn (II): initial mineralogy, oxidation state and ageing. Geochim. Cosmochim. Acta.

[17] Shen XF, Ding YS, Hanson JC, Aindow M, Suib SL (2006). In situ synthesis of mixed-valent manganese oxide nanocrystals: an in situ synchrotron x-ray diffraction study. J. Am. Chem. Soc..

[18] Chen BR (2018). Understanding crystallization pathways leading to manganese oxide polymorph formation. Nat. Commun..

[19] Hem JD, Roberson CE, Fournier RB (1982). Stability of βMnOOH and manganese oxide deposition from springwater. Water Resour. Res..

[20] Hem JD, Lind CJ (1983). Nonequilibrium models for predicting forms of precipitated manganese oxides. Geochim. Cosmochim. Acta.

[21] Shoemaker DP (2014). In situ studies of a platform for metastable inorganic crystal growth and materials discovery. Proc. Natl Acad. Sci..

[22] Jansen M (2002). A concept for synthesis planning in solid‐state chemistry. Angew. Chem. Int. Ed..

[23] Stranski IN, Totomanow D (1933). Rate of formation of (crystal) nuclei and the Ostwald step rule. Z. Phys. Chem..

[24] Navrotsky A (2004). Energetic clues to pathways to biomineralization: precursors, clusters, and nanoparticles. Proc. Natl Acad. Sci..

[25] Sun W, Ceder G (2017). Induction time of a polymorphic transformation. CrystEngComm.

[26] Sun, W., Jayaraman, S., Chen, W., Persson, K. A., & Ceder, G. Nucleation of metastable aragonite CaCO3 in seawater. *Proc. Natl Acad. Sci.* **112**, 20, (2015). 10.1073/pnas.1423898112 PMC4371997 25739963

[27] Kitchaev DA, Ceder G (2016). Evaluating structure selection in the hydrothermal growth of FeS 2 pyrite and marcasite. Nat. Commun..

[28] Nielsen MH, Aloni S, De Yoreo JJ (2014). In situ TEM imaging of CaCO3 nucleation reveals coexistence of direct and indirect pathways. Science.

[29] Navrotsky A (2011). Nanoscale effects on thermodynamics and phase equilibria in oxide systems. ChemPhysChem.

[30] Navrotsky A, Ma C, Lilova K, Birkner N (2010). Nanophase transition metal oxides show large thermodynamically driven shifts in oxidation-reduction equilibria. Science.

[31] Verink, E. D. Simplified procedure for constructing Pourbaix diagrams. *Uhlig’s Corrosion Handbook,* **7***, *111–124 (2011).

[32] Singh AK (2017). Electrochemical stability of metastable materials. Chem. Mater..

[33] Reuter K, Scheffler M (2001). Composition, structure, and stability of RuO 2 (110) as a function of oxygen pressure. Phys. Rev. B.

[34] Hansen HA, Rossmeisl J, Nørskov JK (2008). Surface Pourbaix diagrams and oxygen reduction activity of Pt, Ag and Ni (111) surfaces studied by DFT. Phys. Chem. Chem. Phys..

[35] Zeng Z (2015). Towards first principles-based prediction of highly accurate electrochemical Pourbaix diagrams. J. Phys. Chem. C..

[36] Huang LF, Rondinelli JM (2015). Electrochemical phase diagrams for Ti oxides from density functional calculations. Phys. Rev. B.

[37] Persson KA, Waldwick B, Lazic P, Ceder G (2012). Prediction of solid-aqueous equilibria: scheme to combine first-principles calculations of solids with experimental aqueous states. Phys. Rev. B.

[38] Sun J, Ruzsinszky A, Perdew JP (2015). Strongly constrained and appropriately normed semilocal density functional. Phys. Rev. Lett..

[39] Kitchaev DA (2016). Energetics of MnO 2 polymorphs in density functional theory. Phys. Rev. B.

[40] Tkalych AJ, Yu K, Carter EA (2015). Structural and electronic features of β-Ni (OH) 2 and β-NiOOH from first principles. J. Phys. Chem. C..

[41] Bohn, Hinrich L. "Redox potentials." *Soil Science ***112**: 39-45 (1971).

[42] Sun W, Ceder G (2013). Efficient creation and convergence of surface slabs. Surf. Sci..

[43] Sun W, Ceder G (2018). A topological screening heuristic for low-energy, high-index surfaces. Surf. Sci..

[44] Patra, A., Bates, J. E., Sun, J., & Perdew, J. P. Properties of real metallic surfaces: effects of density functional semilocality and van der Waals nonlocality. *Proc. Natl Acad. Sci.* **114**, E9188-E9196 (2017). 10.1073/pnas.1713320114 PMC5676929 29042509

[45] Birkner N, Navrotsky A (2012). Thermodynamics of manganese oxides: Effects of particle size and hydration on oxidation-reduction equilibria among hausmannite, bixbyite, and pyrolusite. Am. Mineral..

[46] Lindberg RD, Runnells DD (1984). Ground water redox reactions: an analysis of equilibrium state applied to Eh measurements and geochemical modeling. Science.

[47] Fritsch S, Post JE, Navrotsky A (1997). Energetics of low-temperature polymorphs of manganese dioxide and oxyhydroxide. Geochim. Cosmochim. Acta.

[48] Parkhurst, D. L., & Appelo, C. A. J. *User’s guide to PHREEQC (Version 2): computer program for speciation, batch-reaction, one-dimensional transport, and inverse geochemical calculations.* (1999).

[49] Jansen M, Pentin IV, Schön JC (2012). A universal representation of the states of chemical matter including metastable configurations in phase diagrams. Angew. Chem. Int. Ed..

[50] Sun W (2016). The thermodynamic scale of inorganic crystalline metastability. Sci. Adv..

[51] Parsons R (1990). The electrical double layer: recent experimental and theoretical developments. Chem. Rev..

[52] Nemšák S (2014). Concentration and chemical-state profiles at heterogeneous interfaces with sub-nm accuracy from standing-wave ambient-pressure photoemission. Nat. Commun..

[53] Li YF, Zhu SC, Liu ZP (2016). Reaction network of layer-to-tunnel transition of MnO2. J. Am. Chem. Soc..

[54] Reed J, Ceder G (2004). Role of electronic structure in the susceptibility of metastable transition-metal oxide structures to transformation. Chem. Rev..

[55] Birgisson S, Saha D, Iversen BB (2018). Formation mechanisms of nanocrystalline MnO2 polymorphs under hydrothermal conditions. Cryst. Growth Des..

[56] Kresse G, Furthmüller J (1996). Efficient iterative schemes for ab initio total-energy calculations using a plane-wave basis set. Phys. Rev. B.

[57] Kresse G, Joubert D (1999). From ultrasoft pseudopotentials to the projector augmented-wave method. Phys. Rev. B.

[58] Sun J, Ruzsinszky A, Perdew JP (2015). Strongly constrained and appropriately normed semilocal density functional. Phys. Rev. Lett..

[59] Ong SP (2013). Python Materials Genomics (pymatgen): a robust, open-source python library for materials analysis. Comput. Mater. Sci..

[60] Hem, J. D. *Chemical Equilibria and the Rates of Manganese Oxidation*. U.S. Geological Survey–Water Supply Paper 1667A, (1963).

