# Supplementary material for: Non-equilibrium crystallization pathways of manganese oxides in aqueous solution
Source: Nat Commun. 2019 Feb 4;10:573. doi: 10.1038/s41467-019-08494-6 (PMC6362205; doi:10.1038/s41467-019-08494-6)
Supplement: Supplementary file 1 — Supplementary Information [file 41467_2019_8494_MOESM1_ESM.pdf]

Supplementary Table 1. Bulk Formation Energies of Mn-O-H Phases

Formation Energies of Mn-O-H phases in eV/formula unit. For the ions and the lowest-energy phases, we choose the most recent experimental reference when available. For *Fritsch and Navrotsky*, and for *Kitchaev et al*, which provide relative energies between the metastable phases of MnOOH and MnO_2 against the ground state, we reference the formation energies of the metastable phase against the experimental formation energy of Hem and Lind (1983). In this work, we disregard the α, δ, and λ polymorphs of MnO_2, which we previously showed to be stabilized by intercalation of alkali impurity ions.[1] For β-MnOOH, we use the bulk energy as calculated in this work, because the surface energies are calculated from the same structure.

**Phase**	Hem, Lind (1983)[2]	Hem (1978)[3]	Hem (1963)[4]	Fritsch, Navrotsky (1997)[5]	Kitchaev et al. (2015, 2017)[1,6]	Persson et al. (2012)[7]	**Our selection**
Mn^{2+} (aq)	**-2.363**	-2.363	-2.359			-2.387	-2.363
Mn^{3+} (aq)			**-0.850**			-0.850	-0.850
MnO_4^- (aq)						**-4.658**	-4.658
MnO_4^{2-} (aq)			-5.221			**-5.222**	-5.222
$MnOH^+$ (aq)	-4.198	-4.198	-4.198				-4.198
$Mn(OH)_3^-$ (aq)	-7.714						-7.714
$HMnO_2^-$ (aq)	**-5.243**	-5.243	-5.243			-5.243	-5.243
Mn	**0**	0	0				0
MnO			**-3.762**				-3.762
Mn_3O_4	**-13.300**	-13.300	-13.263				-13.300
Mn_2O_3		**-9.132**	-9.100				-9.132
$Mn(OH)_2$ - amorphous		**-6.375**					-6.375
$Mn(OH)_2$ - crystalline	**-6.381**		-6.370				-6.381
β-MnOOH	-5.629				**-5.670**		-5.629
α-MnOOH				**-5.763**	-5.750		-5.763
γ-MnOOH	**-5.780**	-5.780		-5.780	-5.780		-5.780
$Mn(OH)_3$			**-7.849**				-7.849
R-MnO2				-4.765	**-4.767**		-4.767
γ-MnO2	-4.731				**-4.787**		-4.787
β-MnO2	-4.821	-4.821	-4.826	-4.821	**-4.821**		-4.821

Supplementary Note 1: *Ab initio s*tructure prediction for metastable *β*-MnOOH

Feitknechtite, *β*-MnOOH, is a metastable manganese oxyhydroxide often observed during aqueous precipitation. The crystal structure of β-MnOOH is not known, although its XRD pattern has been resolved, and is catalogued as JCPDS card no. 00-018-0804. Because it is often an early precipitate during crystallization, nucleation theory would suggest that *β*-MnOOH has a low surface energy. However, without a crystal structure, we would be unable to construct surface slabs in DFT to compute the surface energy.

To produce a candidate structure for *β*-MnOOH, we perform an *ab initio* crystal structure prediction, by sampling various known layered manganese oxide prototype frameworks, enumerating potential hydrogen positions, and comparing candidate structures until a good match with the known X-ray diffraction profile is attained. For layered manganese oxide prototype frameworks, we chose four general structural frameworks to initialize the structural enumeration, as visualized in Fig. S1:

1.) The O1 stacking of δ-MnO_2 based on pyrochroite $Mn(OH)_2$, but enumerating half occupations of the hydrogen atoms, achieving an MnOOH stoichiometry

2.) The P2 stacking of Birnessite, δ-MnO_2, which has the same layer structure but different layer stackings in the out-of-plane direction. We enumerated hydrogen arrangements in the inter-layer area based on electrostatically-favorable vertices and midpoints of a Voronoi decomposition of the structure, scaled to a 1 Å distance of the nearest oxygen. Note that the P3 stacking seen in, for example, the *β*-NiOOH structure, was obtained as a relaxation of an initial O3 structure.

3.) The O3 stacking of δ-MnO_2, following the same methodology as in 2.) to enumerate hydrogen positions.

4.) The orthorhombic polymorph of $LiMnO_2$, removing the Li atoms and adding hydrogen into the interlayer region by analogy to the lepidocrocite FeOOH structure.

On these four generally layered MnO_2 frameworks, we constructed 42 candidate structures with a MnOOH stoichiometry. All MnOOH candidate structures were then structurally relaxed in SCAN-DFT, considering a ferromagnetic and a representative antiferromagnetic magnetic ordering. The most energetically favorable structures were those with the P2 framework, or a P3 framework obtained as a relaxation of the O3 initial structure, as seen in Supplementary Figures 1 and 2. However, as the energy of the P2 and P3 structures are essentially identical (P3 being more stable by less than 1 meV/atom), we relied on the reference X-ray diffraction pattern to choose between the two structural candidates.

Using the X-ray diffraction calculator in pymatgen, we computed XRD patterns for the candidate structures (with simulated Cu-Kα source), and compared the resulting XRD patterns to the known pattern in JCPDS card no. 00-018-0804.

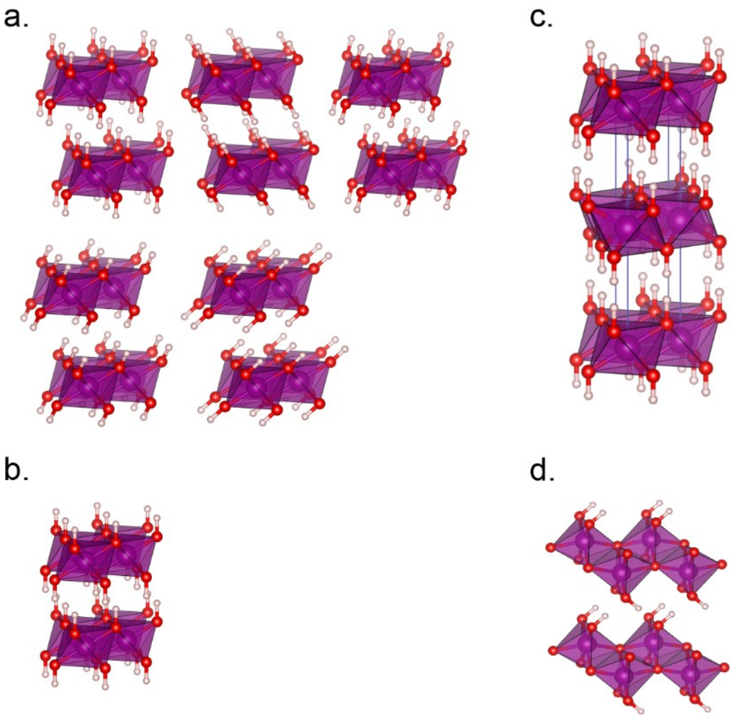

Supplementary Figure 1. Structural frameworks for candidate β-MnOOH structures, where a) O3-type δ-MnO_2, b) O1-type δ-MnO_2 derived from pyrochroite $Mn(OH)_2$, c) P2-type δ-MnO_2 and d) o-$LiMnO_2$-type. The visualized structures in a), b) and c) are in the MnO_2H_2 stoichiometry, with all possible hydrogen positions for MnOOH visualized. Note that the P3 structure, seen in for example *β*-NiOOH, is obtained from a relaxation of the O3 structures in a), in the cases where it is energetically favorable.

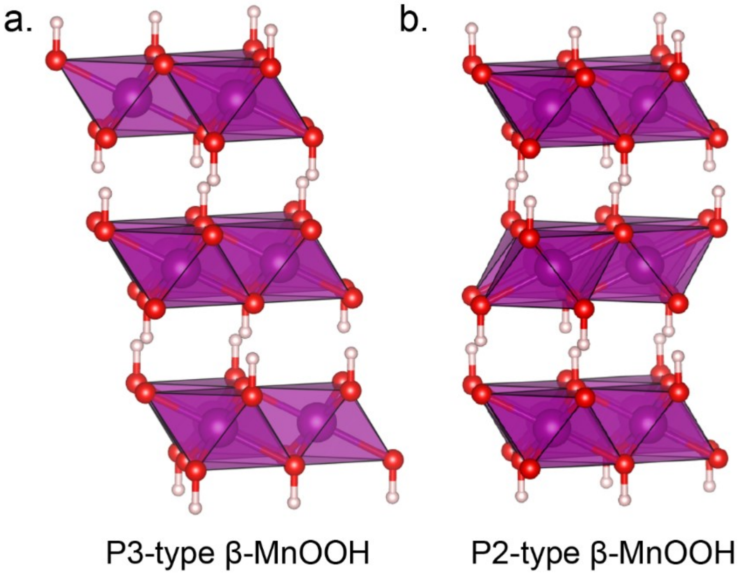

Supplementary Figure 2. Low energy structural candidates for the *β*-MnOOH structure, belonging to the P3 and P2 layered structure types.

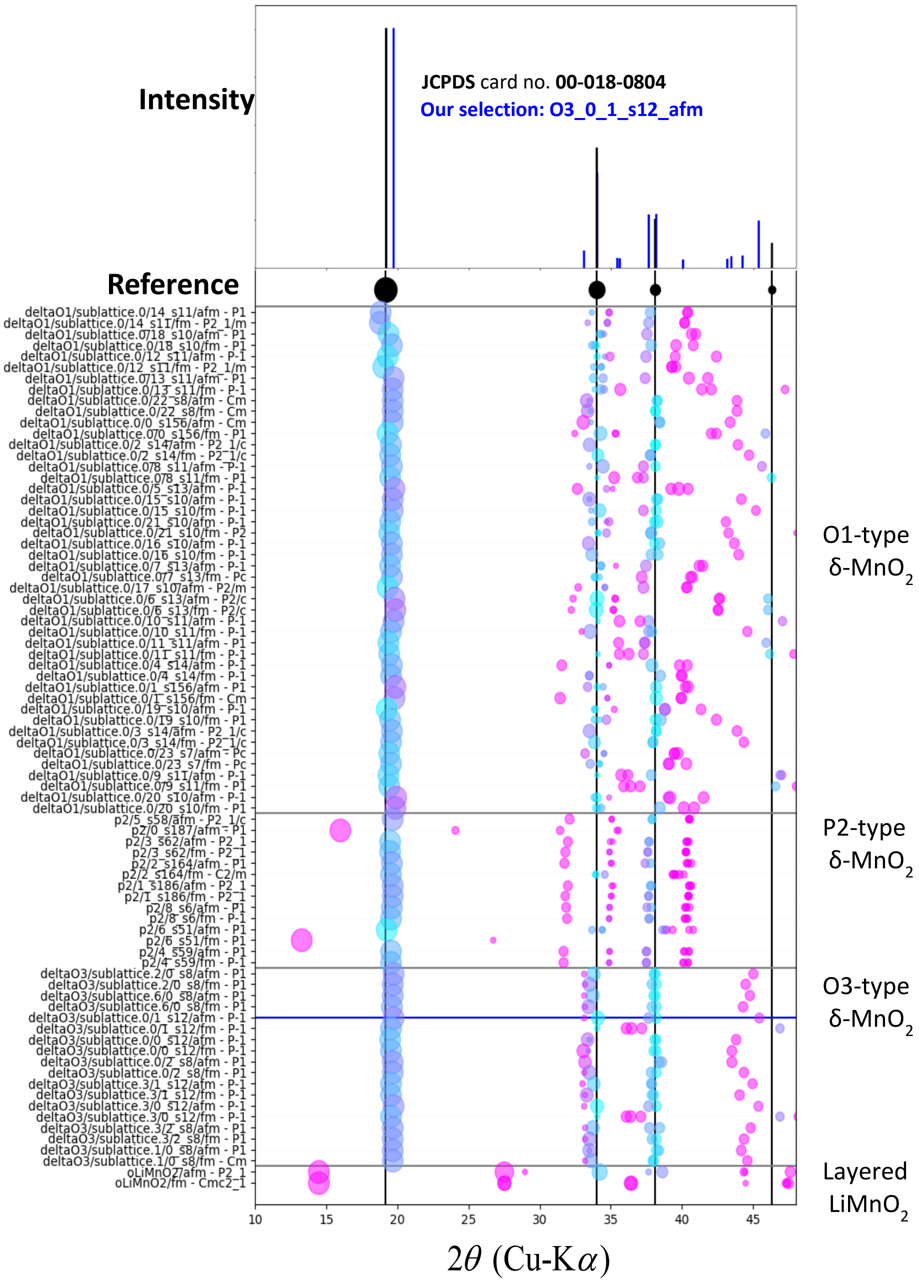

Supplementary Figure 3. Top) X-ray diffraction pattern for both the reference β-MnOOH entry from the JCPDS, and our predicted structure in blue. Bottom) Computed X-ray diffraction patterns for all candidate β-MnOOH structures, with circles centered on the 2θ of the computed peak, and the radius of each circle proportional to the intensity of each peak. The reference XRD pattern is shown as black circles, with corresponding vertical black lines indicating their 2θ position. The color of each circle for the candidate structures is representative of their distance from the nearest reference peak; blue means a reference peak is close by, whereas purple means the nearest reference peak is far away. The horizontal blue line marks the structure of our selected β-MnOOH structure.

Supplementary Figure 3 compares all the XRD patterns for the candidate structures. The $LiMnO_2$ framework has a poor match for the 18° peak of the reference structure, indicating that the layer-spacing is wrong and that this is not the proper framework. The other frameworks; O1-type δ-MnO_2, P2 δ-MnO_2, and O3-derived δ-MnO_2 have the proper first peak position. However, both O1-type δ-MnO_2 and P2 δ-MnO_2 have poor fits on the second and third peaks, suggesting that the out-of-plane vector is at the wrong angle.

Of the four frameworks, P3 δ-MnO_2 (derived as a relaxation of the O3 initial structure) has the best fit on the first three peaks, meaning the β-MnOOH is most likely to exhibit the P3-type framework, with its corresponding out-of-plane vector angle. The Mn^{3+} ions form an antiferromagnetic ordering, as shown in Supplementary Figure 4. The structure is computed to have an enthalpy of ΔH = 8.41 kJ/mol, relative the ground-state γ-MnOOH phase, within typical error tolerance of the experimentally-measured ΔH = 14.6 kJ/mol. This structure is similar to the *β*-NiOOH structure recently computed in Reference 8. The structure file for the DFT-relaxed *β*-MnOOH structure is provided below.

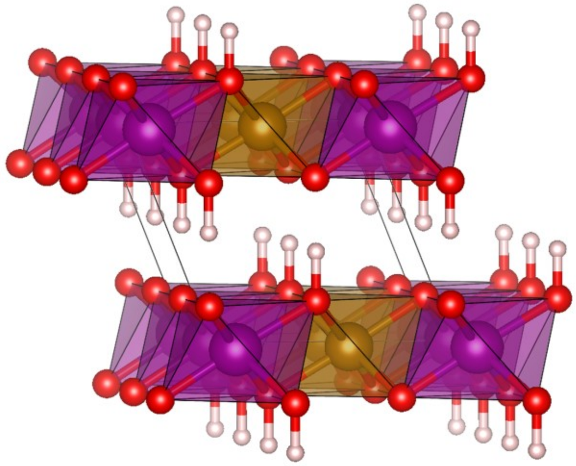

Supplementary Figure 4. Crystal structure of *β*-MnOOH with best XRD fit with JCPDS no. 00-018-0804. Yellow and purple Mn^{3+} ions have alternating magnetic configurations.

VASP POSCAR file for β-MnOOH

```
Mn8 H8 O16
   1.00000000000000
    -1.6113318997295274   -5.1795809311106202    1.5346401243457135
    -5.2080947651807836    2.9836836739942356    6.2484737403908399
    -5.2926840526287560    0.6896720738141182   -3.6157789386391466
   Mn   H    O
     8     8    16
Direct
  0.0056313762313164  0.0001738845426412  0.4919102364441423
  0.5056319105254206  0.5001735736669992  0.9919053999967427
  0.2556262868271123  0.2501747533284313  0.7419080077004379
  0.7556265797173368  0.7501731584947635  0.2419102216138322
  0.4943671178329667  0.4998256433736019  0.5080850516283739
  0.9943688818124320  0.9998259487924266  0.0080915647274343
  0.2443769753272160  0.2498275550742363  0.2580869745728686
  0.7443800554814844  0.7498235288864727  0.7580849762216441
  0.0340832567899250  0.4821203658699273  0.0006144426261998
  0.5340839425119308  0.9821206282508242  0.5006143137979164
  0.4659108347283620  0.0178787003266070  0.9993953454028219
  0.9659125625585587  0.5178786851580480  0.4993942991293925
  0.7159114857557725  0.2678819326361569  0.2493947148024338
  0.2159100680489441  0.7678821992310287  0.7493942780477153
  0.7840853838018385  0.2321166368088614  0.7506137481685543
  0.2840847385100331  0.7321176257277067  0.2506129068750474
  0.9378080699174677  0.1590298314239528  0.2553884332954256
  0.4378093511573214  0.6590283168044663  0.7553862069731374
  0.9345663186960040  0.1580754546573425  0.7562850196342014
  0.4345652544658706  0.6580759795068997  0.2562801376691347
  0.1878088922225290  0.4090305500831429  0.5053910182895607
  0.6878070672413900  0.9090304502833404  0.0053905438182400
  0.1845856006642574  0.4080770433800650  0.0062819791676843
  0.6845858135239654  0.9080769201290961  0.5062831327073700
  0.3121987412590566  0.0909679011165375  0.4946068934232672
  0.8121980604273960  0.5909676297706353  0.9946069348711265
  0.3154109615960788  0.0919219438366433  0.9937153508200250
  0.8154132799437303  0.5919224640961682  0.4937130099934519
  0.5621972720889019  0.3409700076382401  0.7446104250441343
  0.0621960192429241  0.8409706388820181  0.2446126838357809
  0.5654337535696524  0.3419241453922355  0.2437128951956298
  0.0654330875228382  0.8419229028304597  0.7437138535062574
```

Supplementary Note 2: Example Generalized Pourbaix Potential

Here we provide two example Pourbaix Potentials, for the MnO_4^-(aq) ion, and solid γ-MnOOH. The Pourbaix Potential is expressed with units of eV/Mn, as:

$$\overline{\Psi} = \frac{1}{N_M}\left((\mu - N_O \mu_{H_2O}) + (2N_O - N_H)\mu_{H^+} - (2N_O - N_H + Q)E\right)$$

We use μ_{H_2O} to be the experimental formation energy of water to be -2.4576 eV, as from Ref 7.

For the aqueous ion MnO_4^-, the Pourbaix potential is given as

$$\overline{\Psi}_{\mathrm{MnO_4^-}} = \frac{1}{1}\left(\left(-4.658 + RT\ln[\mathrm{Mn}] - (4)(-2.4576\ \mathrm{eV})\right) + \left(2(4) - 0\right)\mu_{H^+} - \left(2(4) - (0) + (-1)\right)E\right)$$

For the solid phase γ-MnOOH, the Pourbaix potential is given as

$$\overline{\Psi}_{\gamma\text{-MnOOH}} = \frac{1}{1}\left((-5.780 - (2)(-2.4576\ \mathrm{eV}) + (2(2) - 1)\mu_{H^+} - (2(2) - (1) + (0))E\right)$$

For nanoscale Pourbaix phase diagrams, the units of the surface energy term are expressed:

$$\left(\frac{1}{R}\right)\eta\rho\gamma\left[\frac{\mathrm{eV}}{\mathrm{Mn}}\right] = \left(\frac{1}{R}\right)\left[\frac{1}{\mathrm{nm}}\right]\cdot\gamma\left[\frac{\mathrm{J}}{\mathrm{m}^2}\right]\cdot\rho\left[\frac{\mathrm{Å}^3}{\mathrm{Mn}}\right]\cdot\eta\left[\frac{\mathrm{m}^2}{(\mathrm{m}^3)^{(2/3)}}\right]\cdot\left(6.24\times10^{-3}\,\frac{\mathrm{eV}\cdot\mathrm{nm}\cdot\mathrm{m}^2}{\mathrm{J}\cdot\mathrm{Å}^3}\right)$$

For the nucleation analyses, the classical nucleation barrier, ΔG^*, is given by:

$$\Delta G^* = \frac{4}{27}\frac{\left(\eta\rho^{2/3}\gamma\right)^3}{\Delta\Psi^2}$$

where γ is the average surface energy of a nucleus, η is a shape-factor (with units Area/$Vol^{2/3}$), and ρ is the volume per manganese atom, in units of $Å^3$/Mn. Therefore, the numerator has units of eV^3/Mn^2, and the denominator has units of eV^2/Mn^2. ΔG* is divided by k_BT in the total nucleation barrier, which is related to the steady-state nucleation rate by

$$J = Z\beta N \exp\left(\frac{-\Delta G^*}{k_B T}\right) \qquad (1)$$

where Z is the Zeldovich Factor, β is the monomer attachment rate to a growing crystal, and *N* is the number of free monomers in solution. *N* will be the same between competing products, and the differences in *Z* and β between metal oxides of similar composition are usually much smaller than differences in the exponential term, which can vary by orders of magnitude. Therefore, analysis of nucleation rates between competing metal oxides can be approximated as having the same exponential pre-factors.[9] If the approximation is invalid, it is straightforward to include kinetic effects in the analysis, by assigning a specific *Z* or β for each phase.

Supplementary Note 3: Surface energies of MnO_xH_y phases

Surface energies were calculated with the equation

$$\gamma = \frac{1}{2A}\left(E_{slab} - NE_{bulk} - \sum_i N_i \mu_i \right)$$

To compute hydrated surface energies, we attempted to model the first monolayer of strongly-bound water by adsorbing explicit water molecules onto the various surfaces. We previously used this technique to successfully model the hydrated surface energies in $CaCO_3$ and FeS_2. However, when we attempt this approach on the manganese oxides, the water molecule dissociates on many MnO_2 surfaces, but not on MnOOH surfaces. The splitting of water into an H^+ and OH^- ion makes us unable to construct a self-consistent water chemical potential for computing a hydrated surface energy using the surface grand potential.

The thermodynamic effect of water hydration is to passivate broken bonds, resulting in a reduction of surface energy. The systematic underbinding of bonds in the generalized-gradient approximation often leads to surface energies being consistently under-predicted from experiment by 30%,[10] which is approximately the same amount as due to hydration. For this reason, we use the SCAN surface energies of *dry* surfaces to approximate the experimental surface energies of *hydrated* surfaces. The dry DFT-calculated surface energies of bixbyite Mn_2O_3 and Pyrolusite β-MnO_2 are found to be within the error bars of the hydrated surface energies, as measured by Birkner and Navrotsky.[11] Therefore, we use the dry surface energies from SCAN to approximate the quantitative value of the hydrated surface energies.

We assume in this work that Δγ between manganese oxide phases does not vary significantly with *E* or *p*H, as chemical interactions between H^+ or OH^- ions and manganese oxide surfaces should be largely similar over changing surface structures. While in reality, H^+ or OH^- ions may adsorb preferentially onto specific surfaces of specific polymorphs,[12] identifying these relationships is enormously expensive computationally, and will be tackled in a future work. We emphasize that this work focuses primarily on the contribution of the thermodynamic framework.

Supplementary Table 2. Calculated surface energies, shape factors, and molar volumes of MnO_xH_y phases

Composition	Surface Energy J/m^2	Shape Factor Area/Vol$^{2/3}$	Volume/Metal Å^3/Mn
$Mn(OH)_2$	0.47	5.69	43.5
Mn_3O_4*	1.43	5.44	26.2
Mn_2O_3	1.36	5.84	26.0
β-MnOOH	0.56	6.40	37.1
α-MnOOH	0.65	7.00	34.7
γ-MnOOH	0.84	6.09	33.5
R-MnO_2	1.33	5.78	29.8
γ-MnO_2	1.44	6.11	28.9
β-MnO_2	1.54	6.35	27.6

Equilibrium particle morphologies are computed using the Wulff construction. For the nucleation barrier, we use a surface energy that is averaged over the particle morphology, computed by the equation

$$\gamma_{\mathrm{Avg}} = \frac{\sum_{hkl} \gamma_{hkl} A_{hkl}}{\sum_{hkl} A_{hkl}}$$

The shape factor, η, is unitless and size-invariant, and is computed from Area/Volume$^{2/3}$ of the Wulff construction. For each structure, we also provide the surface energies of some facets that are slightly too high in energy to appear on the particle morphology, but could potentially be stabilized by adsorbates or variations in solution conditions.

Supplementary Figure 5. Wulff Shape for Pyrolusite, β-MnO_2

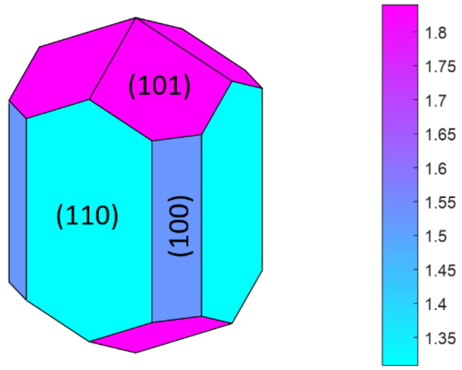

Average surface energy:

1.54 J/m^2

Shape Factor

6.35

Volume/Mn

27.6 Å^3/Mn

Supplementary Table 3. Surface energy data for Pyrolusite, β-MnO_2

Miler Index	Surface Energy (J/m2)	Area Fraction
110	1.31	0.47
101	1.84	0.38
100	1.53	0.15
**Miller Index**	**Surface Energy (J/m2)**	**Energy above Wulff (J/m^2)**
001	2.33	0.126

Supplementary Figure 6. Wulff Shape for Intergrowth, γ-MnO_2

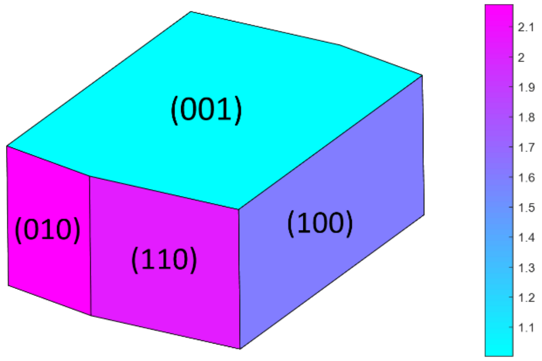

Average SE:

1.44 J/m^2

Shape Factor

6.22

Volume/Mn

28.9 Å^3/Mn

Supplementary Table 4. Surface energy data for Intergrowth, γ-MnO_2

Miler Index	Surface Energy (J/m2)	Area Fraction
001	1.00	0.48
100	1.61	0.29
110	2.06	0.15
010	2.17	0.09
**Miller Index**	**Surface Energy (J/m2)**	**Energy above Wulff (J/m^2)**
111	2.35	0.0889

Supplementary Figure 7. Wulff Shape for Ramsdellite, R-MnO_2

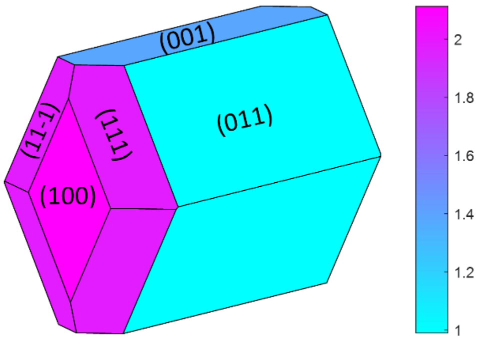

Average SE:
1.33 J/m^2
Shape Factor
5.78
Volume/Mn
29.8 Å^3/Mn

Supplementary Table 5. Surface energy data for Ramsdellite, R-MnO_2

Miler Index	Surface Energy (J/m2)	Area Fraction
011	0.99	0.57
001	1.39	0.17
111	1.99	0.11
11-1	2.00	0.1
100	2.11	0.06

Miller Index	Surface Energy (J/m2)	Energy above Wulff (J/m^2)
110	2.08	0.0146

Supplementary Figure 8. Wulff Shape for γ-MnOOH, Manganite

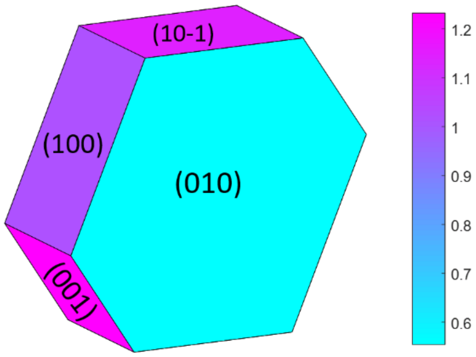

Average SE:
0.84 J/m^2
Shape Factor
6.09
Volume/Mn
33.5 Å^3/Mn

Supplementary Table 6. Surface energy data for Intergrowth, γ-MnO_2

Miler Index	Surface Energy (J/m2)	Area Fraction
010	0.55	0.50
100	1.03	0.20
001	1.15	0.17
10-1	1.23	0.12

Miller Index	Surface Energy (J/m2)	Energy above Wulff (J/m^2)
10-2	1.40	0.0485

Supplementary Figure 9. Wulff Shape for α-MnOOH, Groutite

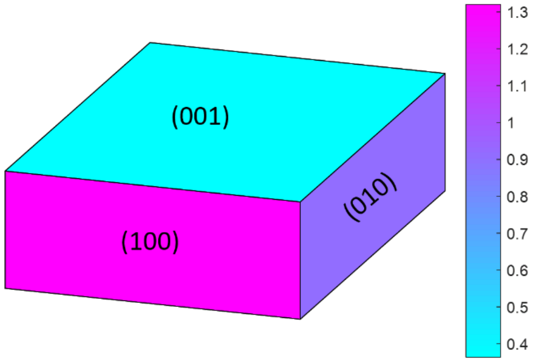

Average SE:
0.65 J/m^2
Shape Factor
7.0
Volume/Mn
34.7 Å^3/Mn

Supplementary Table 7. Surface energy data for Intergrowth, γ-MnO_2

Miler Index	Surface Energy (J/m2)	Area Fraction
001	0.363	0.6
010	0.9239	0.24
100	1.32	0.16

Supplementary Figure 10. Wulff Shape for β-MnOOH, Feitknechtite

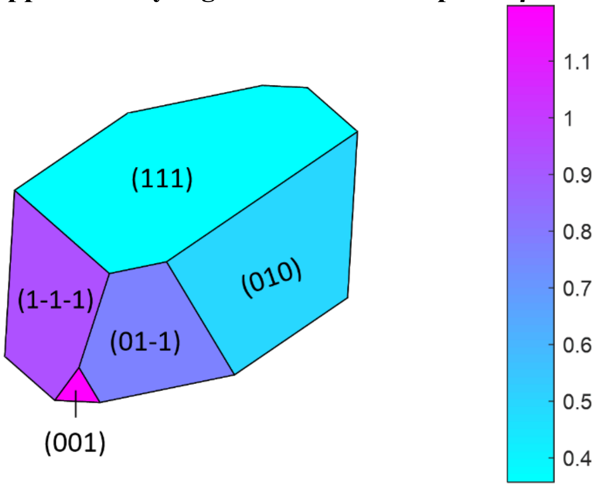

Average SE:
0.53 J/m^2
Shape Factor
6.40
Volume/Mn
37.1 Å^3/Mn

Supplementary Table 8. Surface energy data for β-MnOOH, Feitknechtite

Miler Index	Surface Energy (J/m2)	Area Fraction
111	0.41	0.48
010	0.49	0.26
1-1-1	0.93	0.14
01-1	0.78	0.11
001	1.20	0.006

Miller Index	Surface Energy (J/m2)	Energy above Wulff (J/m^2)
110	1.26	0.29
1-10	1.28	0.3308
100	1.2260	0.3691

Supplementary Figure 11. Wulff Shape for Hausmannite, Mn_3O_4

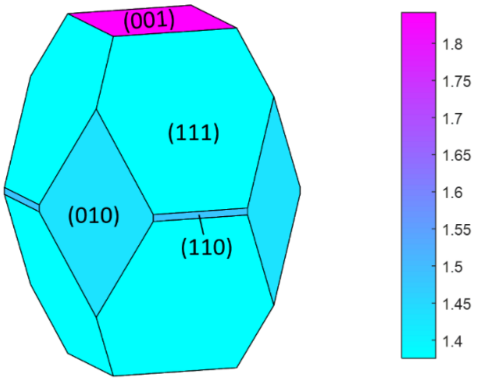

Average SE:
1.43 J/m^2
Shape Factor
5.44
Volume/Mn
26.2 Å^3/Mn

Supplementary Table 9. Surface energy data for Hausmannite, Mn_3O_4

Miler Index	Surface Energy (J/m2)	Area Fraction
111	1.375	0.75
010	1.43	0.13
001	1.84	0.11
110	1.48	0.01

Supplementary Note 4. Polar Surfaces of Hausmannite Mn_3O_4

All surfaces of Hausmannite Mn_3O_4 are polar, and so surface slabs constructed in DFT are subject to the Tasker electrostatic catastrophe,[13] meaning a surface energy cannot be attained without reconstruction. Surface energies reported here are calculated using Tasker 3 to Tasker 2b reconstructions, by moving half of the ions from one side of a slab to the other until the dipole is mitigated. However, such surfaces often result in low-coordination numbers on the surface atoms. This undercoordination results in calculated Mn_3O_4 surface energies that are much higher than experiment, and moreover, do not agree with the general observation that spinel surfaces have low surface energy. The real Hausmannite surface likely exhibits other reconstructions, which may include passivation by the solvent or the electrical double layer, whose structure is beyond the scope of this work. For these reasons, we base the nucleation analyses in this work on the experimentally-measured Hausmannite surface energy of 0.96 J/m^2.

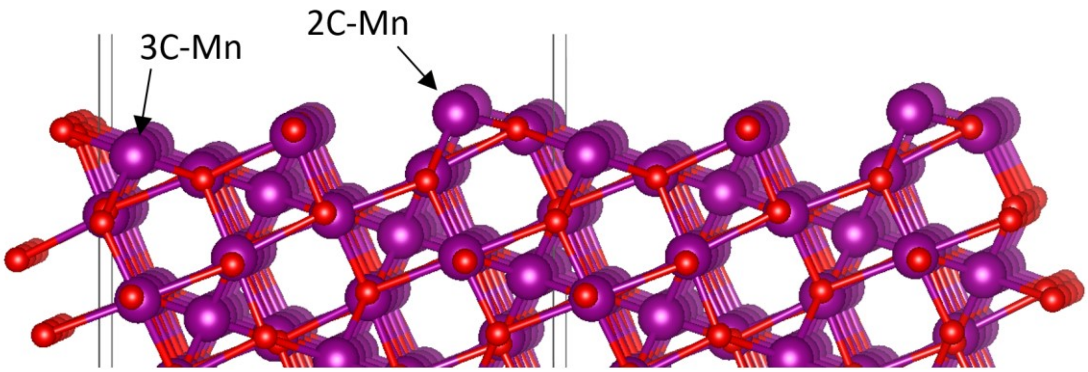

Supplementary Figure 12. Tasker 2B-reconstructed Hausmannite (111) surface, with 2- and 3-fold coordinated surface Mn ions

Supplementary Figure 13. Wulff Shape for Bixbyite, Mn_2O_3

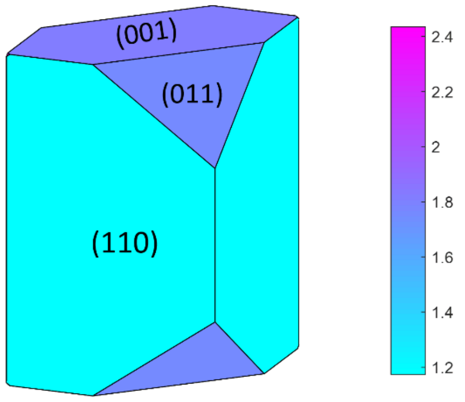

Average SE:

1.36 J/m^2

Shape Factor

5.84

Volume/Mn

26.0 Å^3/Mn

Supplementary Table 10. Surface energy data for Bixbyite, Mn_2O_3

Miler Index	Surface Energy (J/m^2)	Area Fraction
001	1.81	0.18
011	1.77	0.13
110	1.17	0.69
101	2.43	.0001

Miller Index	Surface Energy (J/m^2)	Energy above Wulff (J/m^2)
100	1.81	0.157

Supplementary Figure 14. Wulff Shape for Pyrochroite, $Mn(OH)_2$

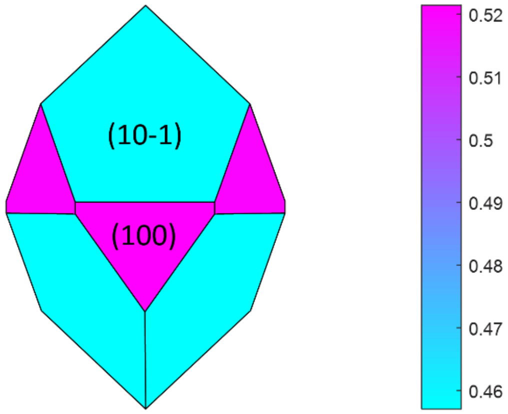

Average SE:

0.47 J/m^2

Shape Factor

5.69

Volume/Mn

43.5 Å^3/Mn

Supplementary Table 11. Surface energy data for Pyrochroite, $Mn(OH)_2$

Miler Index	Surface Energy (J/m^2)	Area Fraction
10-1	0.46	0.79
100	0.52	0.21

Miller Index	Surface Energy (J/m^2)	Energy above Wulff (J/m^2)
110	0.63	0.024
111	0.590	0.014

Supplementary References

[1] Kitchaev, D. A., Dacek, S. T., Sun, W., & Ceder, G. Thermodynamics of phase selection in MnO2 framework structures through alkali intercalation and hydration. *Journal of the American Chemical Society*, *139*(7), 2672-2681. (2017).

[2] Hem, J. D., & Lind, C. J. Nonequilibrium models for predicting forms of precipitated manganese oxides. *Geochimica et Cosmochimica Acta*, *47*(11), 2037-2046. (1983).

[3] Hem, J. D. Redox processes at surfaces of manganese oxide and their effects on aqueous metal ions. *Chemical Geology*, *21*(3-4), 199-218. (1978).

[4] Hem, J. D., "Chemical Equilibria and the Rates of Manganese Oxidation," U.S. Geological Survey-- Water Supply Paper 1667A, 1963

[5] Fritsch, S., Post, J. E., & Navrotsky, A. Energetics of low-temperature polymorphs of manganese dioxide and oxyhydroxide. *Geochimica et Cosmochimica Acta*, *61*(13), 2613-2616. (1997).

[6] Kitchaev, D. A., Peng, H., Liu, Y., Sun, J., Perdew, J. P., & Ceder, G. Energetics of MnO 2 polymorphs in density functional theory. *Physical Review B*, *93*(4), 045132. (2016).

[7] Persson, K. A., Waldwick, B., Lazic, P., & Ceder, G. Prediction of solid-aqueous equilibria: Scheme to combine first-principles calculations of solids with experimental aqueous states. *Physical Review B*, *85*(23), 235438. (2012).

[8] Tkalych, A. J., Yu, K., & Carter, E. A. Structural and electronic features of β-Ni (OH) 2 and β-NiOOH from first principles. *The Journal of Physical Chemistry C*, *119*(43), 24315-24322. (2015).

[9] Sun, W., & Ceder, G. Induction time of a polymorphic transformation. *CrystEngComm*, *19*(31), 4576-4585. (2017).

[10] Goniakowski, J., Holender, J. M., Kantorovich, L. N., Gillan, M. J., & White, J. A. Influence of gradient corrections on the bulk and surface properties of TiO 2 and SnO 2. *Physical Review B*, *53*(3), 957. (1996).

[11] Birkner, N., & Navrotsky, A. Thermodynamics of manganese oxides: Effects of particle size and hydration on oxidation-reduction equilibria among hausmannite, bixbyite, and pyrolusite. *American Mineralogist*, *97*(8-9), 1291-1298. (2012).

[12] Kitchaev, D. A., & Ceder, G. (2016). Evaluating structure selection in the hydrothermal growth of FeS 2 pyrite and marcasite. *Nature communications*, *7*, 13799.

[13] Tasker, P. W. (1979). The stability of ionic crystal surfaces. *Journal of Physics C: Solid State Physics*, *12*(22), 4977.
